# Loss of ZNF451 mediates fibroblast activation and promotes lung fibrosis

**DOI:** 10.1186/s12931-024-02781-7

**Published:** 2024-04-10

**Authors:** Hong Peng, Yu Zhang, Jiali Min, Yuexin Tan, Shanshan Liu

**Affiliations:** 1 Department of Pulmonary and Critical Care Medicine, The Second Xiangya Hospital of Central South University, Changsha, Hunan 410011 China; 2 National Clinical Research Center for Metabolic Diseases, Key Laboratory of Diabetes Immunology, Ministry of Education, Changsha, Hunan 410011 China; 3 Department of Metabolism and Endocrinology, The Second Xiangya Hospital of Central South University, Changsha, Hunan 410011 China

**Keywords:** Pulmonary fibrosis, Fibroblast activation, ZNF451, PDGFB

## Abstract

**Background:**

No effective therapies for pulmonary fibrosis (PF) exist because of the unclear molecular pathogenesis and the lack of effective therapeutic targets. Zinc finger protein 451 (ZNF451), a transcriptional regulator, plays crucial roles in the pathogenesis of several diseases. However, its expression pattern and function in PF remain unknown. This study was designed to investigate the role of ZNF451 in the pathogenesis of lung fibrosis.

**Methods:**

GEO dataset analysis, RT‒PCR, and immunoblot assays were used to examine the expression of ZNF451 in PF; ZNF451 knockout mice and ZNF451-overexpressing lentivirus were used to determine the importance of ZNF451 in PF progression; and migration assays, immunofluorescence staining, and RNA-seq analysis were used for mechanistic studies.

**Results:**

ZNF451 is downregulated and negatively associated with disease severity in PF. Compared with wild-type (WT) mice, ZNF451 knockout mice exhibited much more serious PF changes. However, ZNF451 overexpression protects mice from BLM-induced pulmonary fibrosis. Mechanistically, ZNF451 downregulation triggers fibroblast activation by increasing the expression of PDGFB and subsequently activating PI3K/Akt signaling.

**Conclusion:**

These findings uncover a critical role of ZNF451 in PF progression and introduce a novel regulatory mechanism of ZNF451 in fibroblast activation. Our study suggests that ZNF451 serves as a potential therapeutic target for PF and that strategies aimed at increasing ZNF451 expression may be promising therapeutic approaches for PF.

**Supplementary Information:**

The online version contains supplementary material available at 10.1186/s12931-024-02781-7.

## Introduction

Pulmonary fibrosis (PF) is a chronic interstitial lung disease caused by persistent injuries to the lung parenchyma accompanied by dysregulated lung repair [1, 2]. It is a common pathological change that can be seen in a variety of diseases, including systemic lupus erythematosus, rheumatoid arthritis, systemic sclerosis, and coronavirus disease 2019 (COVID‑19) [3–5]. Idiopathic PF (IPF) is a severe form of lung fibrosis with unknown causes, affecting approximately 5 million people worldwide [6]. IPF has a poor prognosis with a median survival rate of less than 3 years [7]. Although two therapies, pirfenidone and nintedanib, have been approved for the treatment of PF, clinical studies have shown that both can only slow but not stop the progression of PF. There is no effective therapy for PF due to its unclear etiology and pathogenesis. There is an urgent need for new therapeutic approaches for fibrotic lung diseases.

PF is initiated from recurrent injury to the alveolar epithelium, followed by excessive secretion of proinflammatory and profibrotic factors from injured epithelial cells and recruited immune cells that trigger the differentiation of fibroblasts into myofibroblasts [8]. These myofibroblasts, in turn, secrete exaggerated amounts of extracellular matrix (ECM), which results in irreversible lung destruction and loss of respiratory function [9, 10]. Multiple types of cells, including epithelial cells, immune cells, and fibroblasts, have been reported to be involved in the pathogenesis of pulmonary fibrosis [11, 12]. Among them, fibroblasts act as the principal effector cells of fibrosis. Limited treatment options for PF result from the unclear understanding of the molecular mechanisms that regulate the activation of fibroblasts into myofibroblasts.

ZNF451, a poorly characterized vertebrate zinc-finger protein, was initially identified as a SUMO2/3-specific E3 ligase [13–15]. In addition to its function as an E3 ligase that regulates the stability of its SUMO substrates, such as TWIST and PML, ZNF451 is also known as a transcriptional cofactor [16, 17]. For instance, Karvenon et al. found that ZNF451 acts as a transcriptional coactivator for the androgen receptor (AR), leading to the enhanced expression of AR target genes [13]. Feng et al. revealed that ZNF451 acts as a transcriptional corepressor for Smad3/4 and thus negatively regulates TGF-β signaling [18]. We recently identified ZNF451 as a transcriptional coactivator for SLUG, a key regulator of epithelial-mesenchymal transition, thus facilitating SLUG-mediated CCL5 transcription [19]. The role of ZNF451 in the pathogenesis of pulmonary fibrosis is unknown.

In the present study, we identified ZNF451 as a key regulator of fibroblast activation in PF progression. ZNF451 was expressed at low levels and associated with disease severity in PF. The overexpression of ZNF451 markedly alleviated, while the loss of ZNF451 aggravated, BLM-induced pulmonary fibrosis. Mechanistically, the reduced expression of ZNF451 increased the expression of PDGFB, which triggered the PI3K/Akt signaling pathway to enhance fibroblast activation. Our findings present ZNF451 as a therapeutic target for the treatment of PF.

## Materials and methods

### Collection of human lung tissues

Fresh human fibrotic lung specimens were obtained from PF patients undergoing lung transplantation. Fresh human normal lung specimens were obtained at least 10 cm away from the lesion in patients undergoing surgery for pulmonary nodules. All human specimens were obtained from the Second Xiangya Hospital of Central South University. Protocols using human specimens were approved by the Ethics Committee of National Clinical Research Center of the Second Xiangya Hospital of Central South University (approved no. 2022-052). Informed consent was obtained from all subjects. The study conformed to the principles outlined in the Declaration of Helsinki.

### Cell culture

MRC5 cells were cultured in minimal essential medium (Thermo Fisher) with 10% FBS, penicillin, streptomycin, and nonessential amino acids (NEAA) and maintained at 37 °C and 5% CO_2_.

### Animal studies

C57BL/6J male mice (8 weeks old) were purchased from SJA Laboratory Animal Co., Ltd. (Hunan, China). *Znf451*-/- mice were constructed by Cyagen Biosciences Inc. (Suzhou, China). Mice were bred and housed under specific pathogen-free conditions at the Animal Care Center in the Second Xiangya Hospital of Central South University. All mouse experiments were approved by the Ethics Committee of the Second Xiangya Hospital of Central South University. Mice were randomly assigned to experimental groups. Treatment groups were blinded, and no outliers were excluded from the datasets.

### Bleomycin-induced mouse PF model

The bleomycin (BLM)-induced mouse PF model was generated by repetitive intratracheal BLM spray as previously described [20]. Briefly, mice (C57Bl/6J, 9–10 weeks of age) were anesthetized with Avertin (Sigma), 40 mg/kg i.p., intubated, and given an intratracheal instillation of 1 U/kg BLM (Selleck) or an equivalent volume of saline for a total of 6 times, with 14-day intervals between each instillation. Mice were sacrificed by excessive anesthesia 10 days after the last BLM challenge.

### Isolation of primary mouse lung fibroblasts

Primary lung fibroblasts were isolated from mice as previously reported [21]. After the fresh lung tissue of mice treated with or without BLM was chopped, the tissue was cut into 1 mm^3^ pieces in DMEM. After centrifugation at 1,500 rpm for 10 min, the tissue suspension was suspended in DMEM containing 15% FBS and spread evenly in a 10-cm dish. After 4–5 days of culture, the adherent fibroblasts were harvested for passage or for assays. Primary lung fibroblasts were cultured for no more than 3 passages.

### Immunofluorescence staining

Cells cultured on coverslips were fixed with 4% (v/v) formaldehyde at room temperature for 15 min and permeabilized with 0.5% Triton X-100 at room temperature for 20 min. After blocking with 3% BSA at room temperature for 30 min, the cells were incubated with antibodies against alpha smooth muscle actin (α-SMA) at 4 °C overnight, followed by staining with Alexa Fluor 594-conjugated anti-mouse antibodies at room temperature. For visualization of F-actin, cells were stained with Alexa Fluor™ 488-phalloidin for 15 min at room temperature. The sections were mounted with 4,6-diamidino-2-phenylindole (DAPI)-containing mounting medium and imaged using a Zeiss LSM 780 Laser Scanning Confocal Microscope.

### Immunoblotting

Mouse lung tissues or cells were lysed with RIPA Lysis Extraction Buffer (Beyotime Technology) along with protease inhibitor cocktail (Selleck). The total protein concentration was determined by a BCA protein assay reagent kit (Applygen Technologies Inc.) according to the manufacturer’s protocol. Total protein (20 µg) was loaded and separated by 10% SDS‒PAGE and then transferred to a PVDF membrane. Membranes were blocked with 5% skim milk in TBST buffer for 1 h and then incubated with primary antibodies overnight at 4 °C, followed by incubation with horseradish peroxidase-conjugated secondary antibodies. The following antibodies were used: anti-ZNF451 (Proteintech), anti-α-SMA (BOSTER), anti-Col1 (Abcam), and anti-GAPDH (ZSGB BIO). The signaling was visualized using a ChemiDocTM XRS + with Image LabTM Software (Bio-Rad, Hercules, California, USA) with an ECL kit (Tanon).

#### RNA isolation and quantitative RT‒PCR

Total RNA from cells was prepared using the EastepTM Super Total RNA Extraction Kit (Promega, Beijing, China), and cDNA was synthesized using EasyScript one-step gDNA Removal and cDNA Synthesis SuperMix (TransGen, Beijing, China). qRT‒PCR analyses were performed using 2X qPCR Master Mix (KAPA BIOSYSTEMS, Wilmington, Massachusetts, USA) and Applied Biosystems® ViiA™ Real-Time PCR System (Applied Biosystems, Carlsbad, CA, USA). All reactions were carried out in triplicate. PCR primer sequences for qRT‒PCR are listed in Table S1.

### Cell migration assay

Fibroblasts were cultured in serum-free DMEM for 24 h prior to cell migration assays. This assay was performed using transwell inserts (8 μm pore size) in 24-well culture plates. Cells were seeded (5 × 10^5^ cells/mL) into the upper chamber in FBS-free medium, while the lower chamber contained medium with 10% FBS. Normal IgG or PDGFB antibody was added to the lower chamber. After 12 h, the medium was removed, and the migrated cells in the lower chamber were stained with crystal violet. Invasive cells from 3 nonoverlapping fields of each membrane were imaged and counted using a brightfield microscope (Olympus IX71, Olympus Optical, Tokyo, Japan) with a ×10 objective.

### Oris™ pro cell migration assay

This assay was performed with an Oris cell migration assay kit (Platypus Technologies, Fitchburg, WI, USA) according to the manufacturer’s instructions. Briefly, primary mouse lung fibroblasts were seeded (2.5 × 10^4^ cells/well) in an Oris Pro Cell Migration Assay 96-well tissue culture-treated plate. Two hours later, once the cells were adhered to the plates, the plate was incubated for an additional 12 h to allow cells to migrate. The wells were then washed with PBS and fixed with 4% paraformaldehyde (PF) for 15 min. After being washed 3 times, the wells were incubated with CoraLit® 594-phalloidin for 2 h at room temperature. Images were captured with a confocal microscope (TCS SP2, Leica Microsystems, Heidelberg, GmbH) utilizing a 4× objective.

### Hydroxyproline assay

The collagen content was measured using a hydroxyproline assay kit (NanJing JianCheng Bioengineering Institute) according to the manufacturer’s instructions. Briefly, mouse lung tissues were hydrolyzed by adding alkaline acid at 95 °C for 20 min. Then, the pH was adjusted to 6.0-6.8 using the reagent provided. Finally, active carbon was added to each sample, and the supernatant was carefully taken for measurement using an HTS 7000 Plus Bio Assay Reader (Perkin Elmer). Hydroxyproline content in each sample was calculated as ‘ug per right lung’.

### Lung function measurement

Mice were placed on a flexiVent pulmonary system (SCIREQ Inc., Montreal, Canada) under anesthesia. Mice were mechanically ventilated with a tidal volume of 10 ml/kg, a positive end expiratory pressure (PEEP) of 3 cmH_2_O, and a ventilatory frequency of 150 breaths/min. Static compliance (Cst) was recorded for endpoint measurements.

### Lentivirus transduction

For lentivirus administration, lentiviruses (5 × 10^7^ I.U.) overexpressing *Znf451* in 50 µl of PBS were administered to mice via intratracheal instillation for a total of two treatments at 2-week intervals beginning on day 10 after the last BLM administration.

### Generation of stable cell lines

To generate MRC5 cells stably expressing *ZNF451*-shRNA or the Ctrl-shRNA, lentivirus were infected into MRC5 cells in the presence of 5 µg/mL polybrene. After 48 h of infection, cells were selected in medium containing 1 µg/ml puromycin (Gibco, CA, USA).

### RNA-Seq

Total RNA from MRC5^CTRL^ and MRC5^ZNF451KD^ cells was extracted using TRIzol reagent (Invitrogen, CA, USA) according to the manufacturer’s protocol. RNA purity and quantification were evaluated using a NanoDrop 2000 spectrophotometer (Thermo Scientific, USA). RNA integrity was assessed using an Agilent 2100 Bioanalyzer (Agilent Technologies, Santa Clara, CA, USA). Then, the libraries were constructed using the VAHTS Universal V6 RNA-seq Library Prep Kit according to the manufacturer’s instructions. Transcriptome sequencing and analysis were conducted by OE Biotech Co., Ltd. (Shanghai, China). The SRA accession number is PRJNA963236 in this study.

### Statistical analysis

Data are expressed as the mean ± standard error of the mean (SEM). Statistical significance was evaluated by Student’s t test or one-way ANOVA. Differences between groups were significant at a P value of < 0.05. Statistical analyses were performed with GraphPad Prism 9 (GraphPad Software, Inc., San Diego, CA).

## Results

### ZNF451 is downregulated and negatively associated with disease severity in IPF

**Fig. 1:**
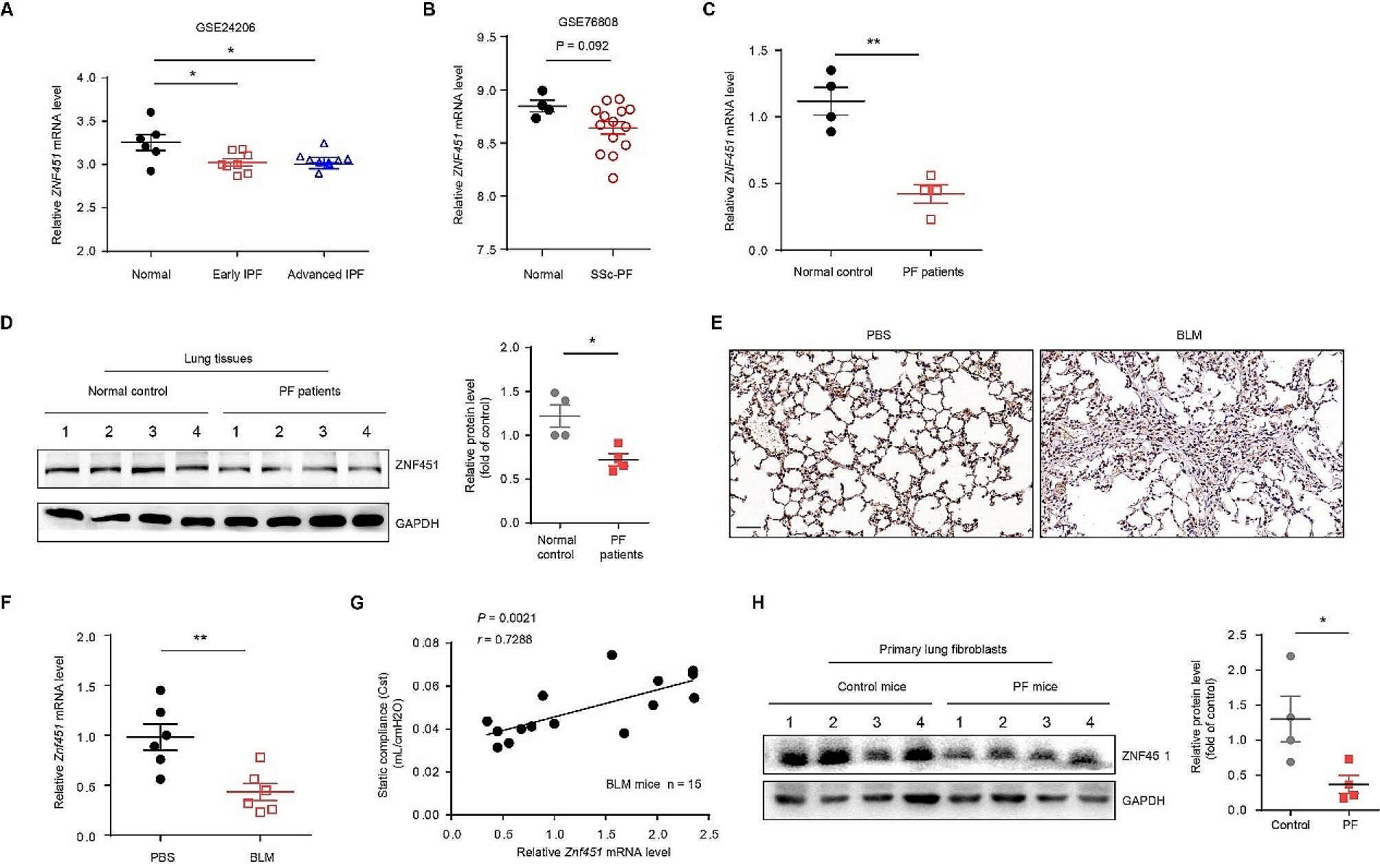
Expression of ZNF451 is decreased in PF lungs and is negatively associated with PF. **A** Scatter plots of *ZNF451* expression in lung tissues of patients with IPF and normal controls from a public microarray dataset (GSE24206) (normal, *n* = 6; early IPF, *n* = 8; advanced IPF, *n* = 9). **B** Scatter plots of *ZNF451* expression in lung tissues of patients with SSc-PF and normal controls from a public microarray dataset (GSE76808) (normal, *n* = 4; SSc-PF, *n* = 14). **C** RT‒PCR of *ZNF451* mRNA in lung tissues of patients with PF and controls (control, *n* = 4; PF, *n* = 4). **D** Immunoblots of ZNF451 in lung tissues from PBS- or BLM-challenged mice (PBS mice, *n* = 4; BLM mice, *n* = 4). **E** Representative images showing ZNF451 IHC in lung tissues from control mice and BLM-induced PF mice. Scale bars, 50 μm. **F** RT‒PCR of *Znf451* mRNA in lung tissues from PBS- or BLM-challenged mice (PBS mice, *n* = 6; BLM mice, *n* = 6). **G** Correlation analysis between *Znf451* expression in the lungs and static compliance of the respirable system (Cst) in mice. Each point represents the value of one mouse. Spearman’s rank correlation test was employed to determine statistical significance (BLM, *n* = 15). **H** Immunoblots of ZNF451 in primary lung fibroblasts from PBS- or BLM-challenged mice (*n* = 4 per group)

To explore ZNF451 expression in the context of PF, we analyzed the public dataset GSE24206 (early IPF = 8, advanced IPF = 9, control = 6) and found decreased *ZNF451* expression in the lungs of both early IPF and advanced IPF patients compared to controls (Fig. 1A). Reanalysis of an additional study (GSE76808) demonstrated a decreased ZNF451 mRNA level in the lungs of Scleroderma-associated pulmonary fibrosis (SSc-PF) patients (*n* = 14) compared to that of controls (*n* = 4) (Fig. 1B). We further analyzed *ZNF451* expression in lungs from PF patients and controls using RT‒PCR. RNA levels declined in PF lungs compared with controls (Fig. 1C). Consistent results were also obtained by western blot analysis of ZNF451 expression (Fig. 1D). We examined ZNF451 expression in lung tissues from BLM-induced PF mice using immunohistochemistry (IHC). As shown in Fig. 1E, ZNF451 was significantly decreased in samples from PF mice compared to those from control mice. Similarly, lungs from mice following BLM induction also exhibited lower *Znf451* mRNA levels than that of controls (Fig. 1F). Moreover, decreased *Znf451* in PF lungs was associated with reduced pulmonary function, based on static compliance of the respirable system (Cst) (Fig. 1G). Western blot further showed that ZNF451 protein levels declined in primary lung fibroblasts from PF mice compared with controls (Fig. 1H). These data suggest that pulmonary fibrosis is characterized by decreased expression of ZNF451 along with reduced pulmonary function.

### ZNF451 knockout exacerbates BLM-induced PF in mice

**Fig. 2:**
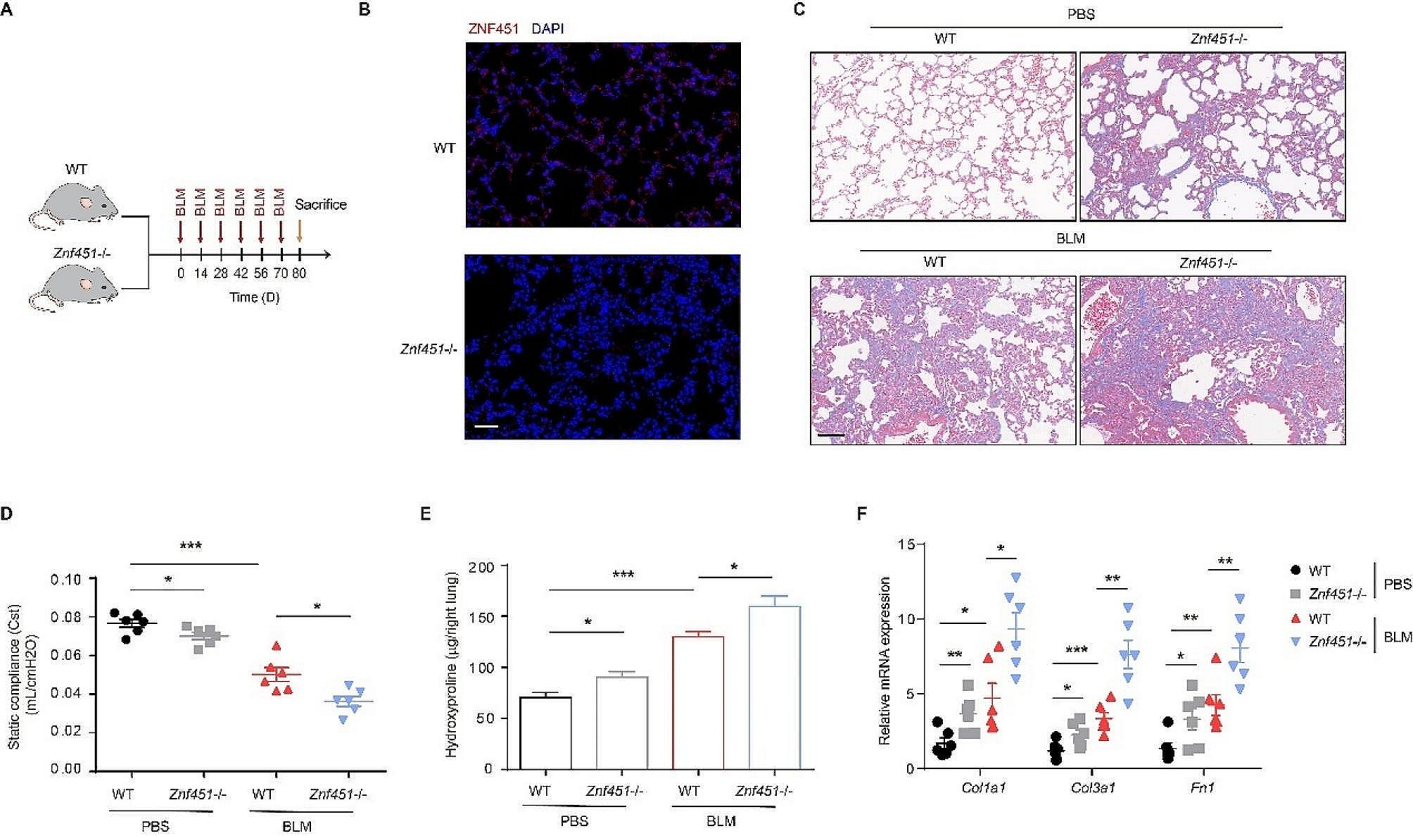
Genetic deletion of ZNF451 attenuates BLM-induced PF. **A** Schematic overview of the experimental design for (C-F). **B** Immunofluorescence staining of ZNF451 in lung tissue sections from WT or *Znf451*-/- mice. Scale bars, 50 μm. **C** Masson staining was performed to evaluate fibrotic changes in PBS- or BLM-challenged WT mice or *Znf451*-/- mice (*n* = 6 per group). Scale bars, 100 μm. **D** Cst was measured to evaluate the lung function of PBS- or BLM-challenged WT mice or *Znf451*-/- mice. **E** Hydroxyproline content in PBS- or BLM-challenged WT mice or *Znf451*-/- mouse lungs. **F** RT‒PCR of mRNA expression of fibrosis genes in PBS- or BLM-challenged WT mice or *Znf451*-/- mice lungs

To explore the role of ZNF451 in PF, saline or BLM (1 U/kg) was intratracheally instilled into *Znf451−/−* and wild-type (WT) mice (Fig. 2A). Immunofluorescence staining showed that WT mice displayed high ZNF451 expression, whereas *Znf451−/−* mice lacked ZNF451 expression (Fig. 2B). Masson’s trichrome staining showed that BLM induced a significant increase in collagen deposition in *Znf451−/−* mice compared with WT mice (Fig. 2C). Cst was further measured to evaluate lung function in these mice in response to BLM. The results in Fig. 2D show that the Cst of the *Znf451−/−* mice were significantly lower than those of the WT control mice in response to BLM. Likewise, the higher severity of fibrosis in *Znf451−/−* mice was characterized by higher levels of hydroxyproline than that in WT mice (Fig. 2E). qPCR assays further revealed that the mRNA levels of fibrotic-related genes, including *Col1a1*, *Col3a1*, and *Fn1*, were significantly increased in BLM-exposed *Znf451−/−* mice compared with BLM-exposed WT mice (Fig. 2F). These results indicate that ZNF451 deficiency aggravated BLM-induced PF.

### ZNF451 overexpression mitigates BLM-induced PF in mice

**Fig. 3:**
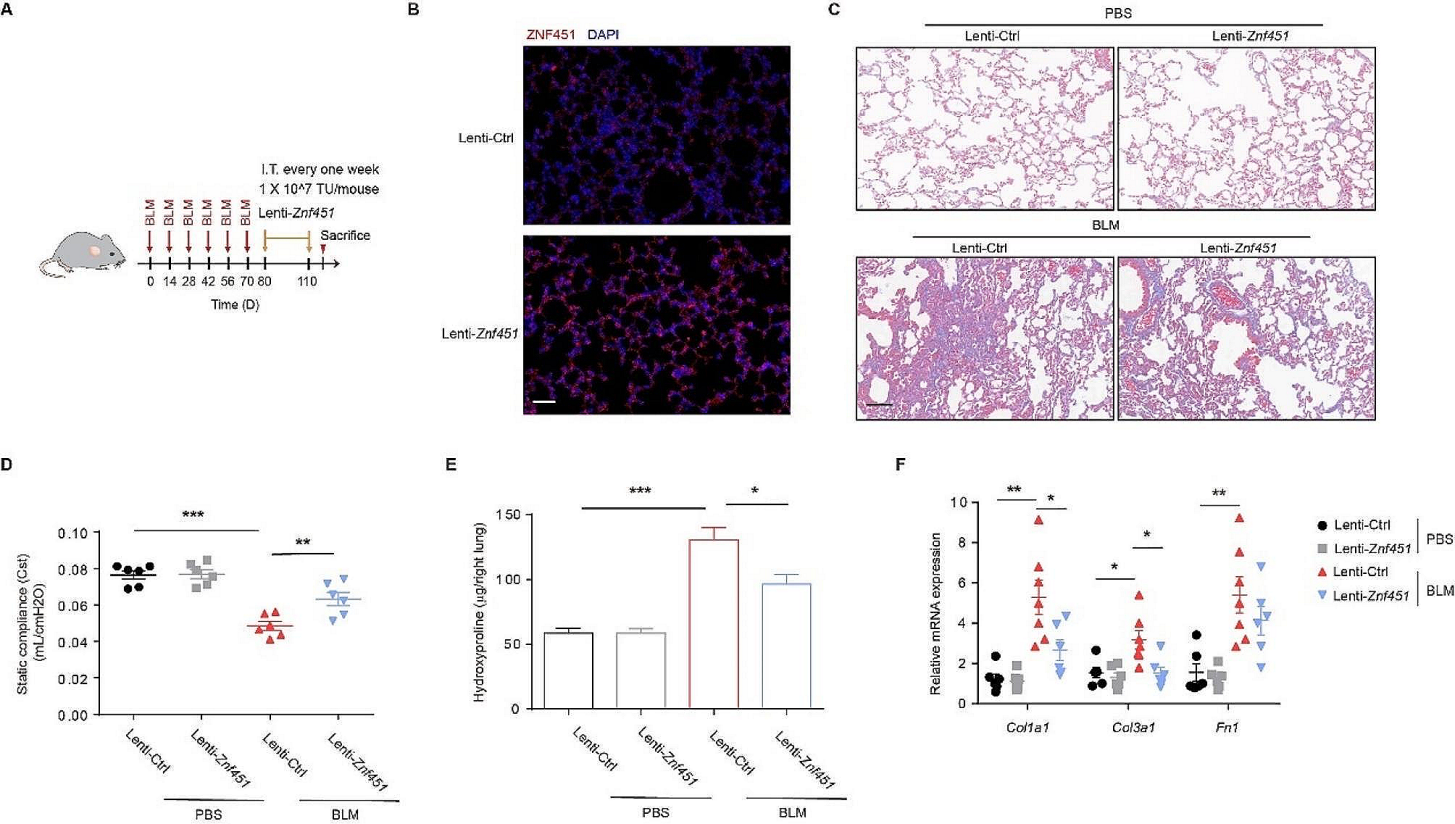
ZNF451 overexpression aggravates BLM-induced PF. **A** Schematic overview of the experimental design for (C-F). **B** Immunofluorescence staining of ZNF451 in lung tissue sections from Lenti-Ctrl- or Lenti-*Znf451*-treated mice. Scale bars, 50 μm. **C** Masson staining was performed to evaluate fibrotic changes in the indicated mice (Group 1: PBS + Lenti-Ctrl, *n* = 6; Group 2: PBS + Lenti-*Znf451*, *n* = 6; Group 3: BLM + Lenti-Ctrl, *n* = 7; Group 44: BLM + Lenti-*Znf451*; *n* = 6). Scale bars, 100 μm. **D** Cst was measured to evaluate the lung function of BLM-exposed mice after *Znf451* overexpression. **E** Hydroxyproline content in lungs from BLM-exposed mice after *Znf451* overexpression. **F** RT‒PCR of mRNA expression of fibrosis genes in lungs from BLM-exposed mice after *Znf451* overexpression

To further confirm the role of ZNF451 in the pathogenesis of pulmonary fibrosis, we instilled *Znf451*-overexpressing lentivirus into lung tissue 10 days after the last BLM challenge (Fig. 3A). Enhanced ZNF451 expression was observed in lung tissues of Lenti-*Znf451*-treated mice (Fig. 3B). The degree of fibrosis in mice was significantly reduced in mice with *Znf451* overexpression, as revealed by the reduced collagen deposition (Fig. 3C), and this result was further confirmed by the improved lung function and decreased hydroxyproline levels in the *Znf451*-overexpressing mice (Fig. 3D, E). The mRNA expression of the fibrotic-related genes *Col1a1*, *Col3a1*, and *Fn1* was also significantly decreased in the lungs of mice instilled with *Znf451*-overexpressing lentivirus (Fig. 3F). Together, these data suggest that ZNF451 overexpression promotes fibrosis resolution in BLM-induced PF model.

#### ZNF451 acts as a repressor of lung fibroblast activation

**Fig. 4:**
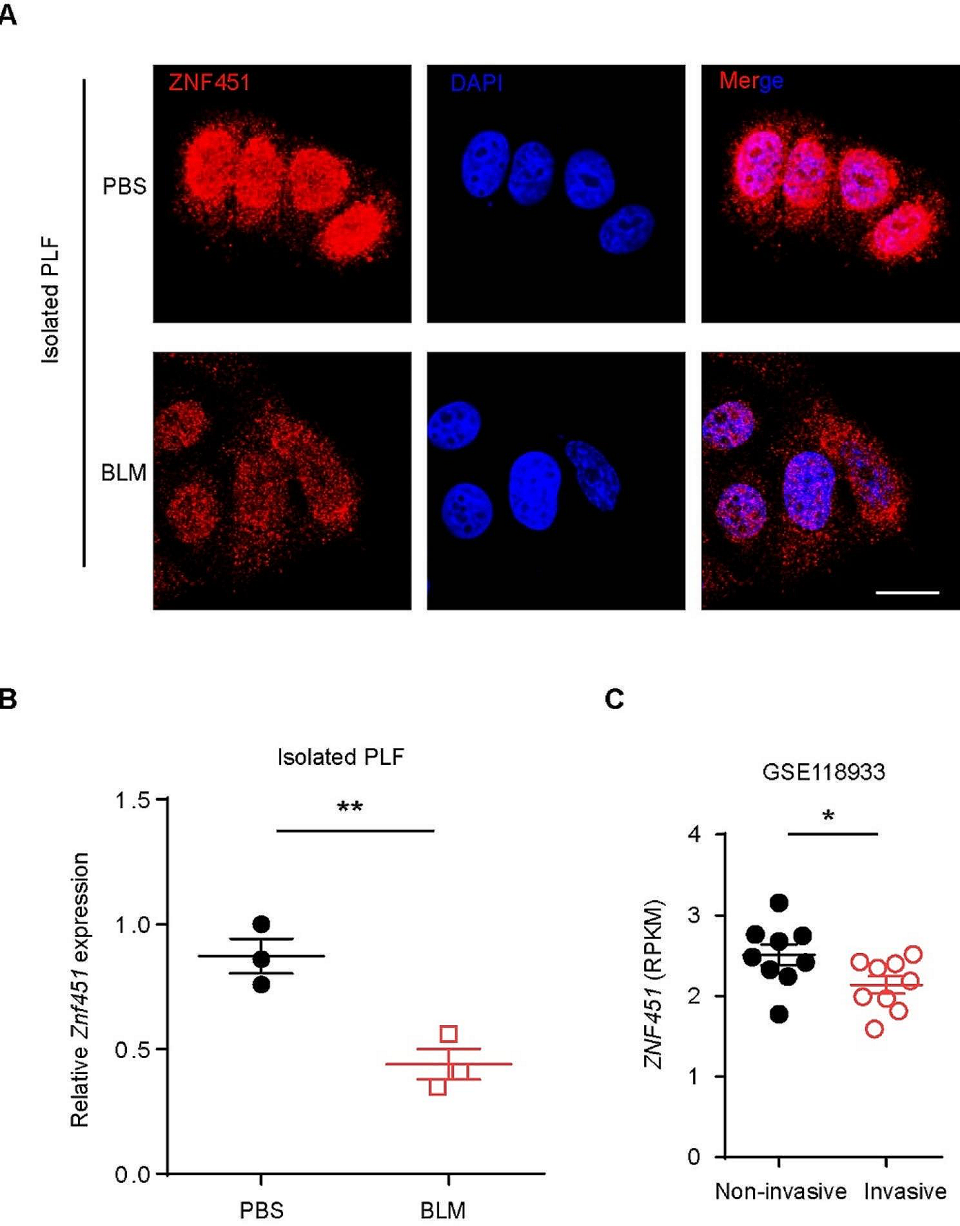
ZNF451 levels were significantly decreased in fibroblasts from PF lungs compared with controls. **A** Immunofluorescence staining of ZNF451 in fibroblasts from BLM-induced PF lungs and controls. Scale bars, 10 μm. **B** RT‒PCR of *Znf451* mRNA in fibroblasts from BLM-induced PF lungs and controls. **C** Scatter plots of *ZNF451* expression in noninvasive and invasive lung fibroblasts from patients with IPF from a public microarray dataset (GSE118933) (noninvasive, *n* = 9; invasive, *n* = 9)

The above results prompted us to explore the mechanism by which ZNF451 mediates pulmonary fibrosis. Given that lung fibroblasts act as the principal effector cells in PF progression, we examined the expression of ZNF451 in the lung fibroblasts of mice following BLM-induced fibrosis. Immunofluorescence analysis showed that ZNF451 was downregulated in lung fibroblasts from BLM-induced PF mice compared to saline-treated control mice (Fig. 4A). Consistently, the mRNA expression of *Znf451* was significantly reduced in the lung fibroblasts of PF mice, as shown by qPCR analysis (Fig. 4B). In addition, reanalysis of a public dataset, GSE118933, revealed that ZNF45 was expressed at low levels in invasive IPF fibroblasts compared with noninvasive IPF fibroblasts (Fig. 4C).

**Fig. 5:**
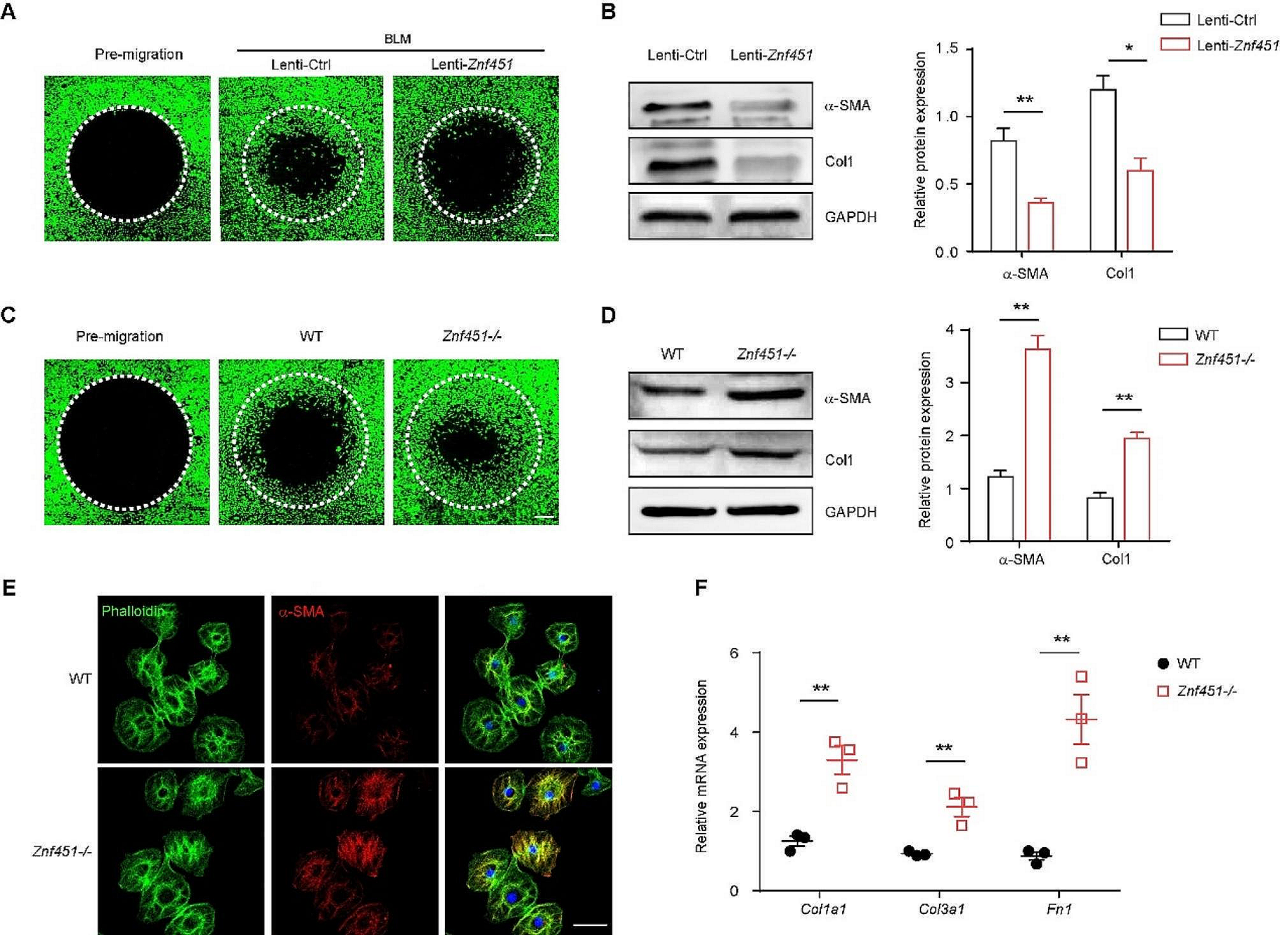
ZNF451 regulates the activation of lung fibroblasts. **A** Representative images of the migration assay for mouse PF lung fibroblasts with or without ZNF451 overexpression. Scale bars, 200 μm. **B** Immunoblots of α-SMA and Col1 in mouse PF lung fibroblasts with or without ZNF451 overexpression. **C** Representative images of the migration assay for mouse lung fibroblasts isolated from WT or *Znf451*-/- mice. Scale bars, 200 μm. **D** Immunoblots of α-SMA and Col1 in mouse lung fibroblasts isolated from WT or *Znf451*-/- mice. **E** Representative images of α-SMA immunostaining in mouse lung fibroblasts isolated from WT or *Znf451*-/- mice. Scale bars, 50 μm. **F** RT‒PCR of the mRNA expression of fibrosis genes in mouse lung fibroblasts isolated from WT or *Znf451*-/- mice

We then examined whether the dysregulation of ZNF451 in fibroblasts contributes to fibroblast activation. Primary lung fibroblasts were isolated from BLM-challenged mice with or without ZNF451 overexpression. The cell migration assay showed that overexpression of ZNF451 significantly suppressed the migration ability of primary lung fibroblasts (Fig. 5A). Consistently, western blot analysis also verified reductions in the expression of the myofibroblast markers α-SMA and Col1 in ZNF451-overexpressing lung fibroblasts (Fig. 5B).

Primary lung fibroblasts were then isolated from WT or *Znf451*-/- mice. The *Znf451*-silenced cells exhibited an enhanced capacity for cell migration (Fig. 5C) and increased expression of the myofibroblast markers α-SMA and Col1 (Fig. 5D). In addition, silencing *Znf451* in lung fibroblasts induced an increase in the percentages of α-SMA-positive cells (Fig. 5E). Consistent results were also obtained by RT‒PCR analysis of the expression of fibrotic-related genes, including *Col1a1*, *Col3a1*, and *Fn1* (Fig. 5F). These results indicate that the loss of ZNF451 during PF pathogenesis contributes to the activation of fibroblasts.

#### ZNF451 deficiency promotes fibroblast activation by increasing the secretion of PDGFB

**Fig. 6:**
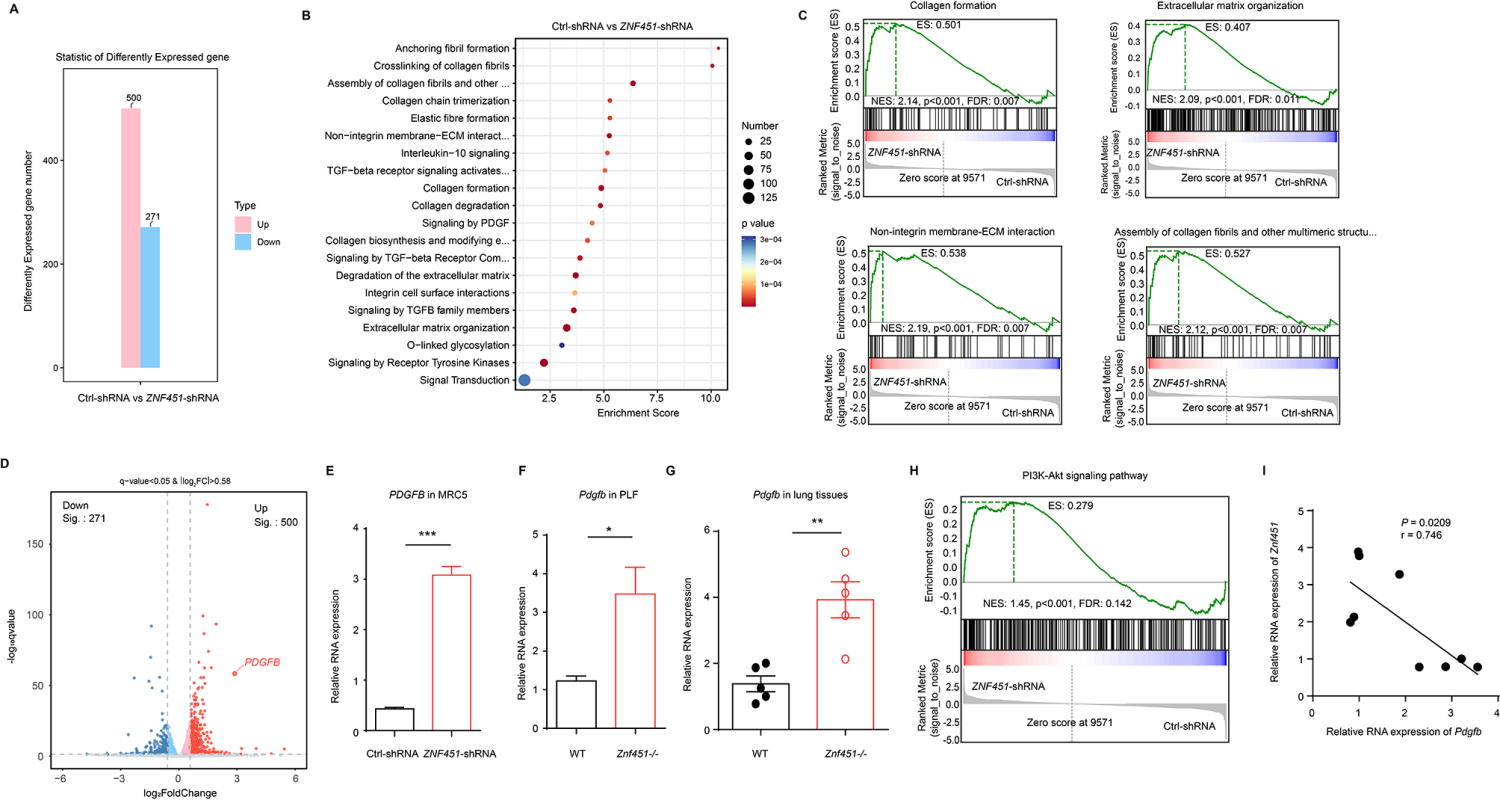
ZNF451 depletion increases PDGFB expression. **A** Bar graph showing differential gene expression between Ctrl-shRNA and *ZNF451*-shRNA MRC5 cells. **B** Reactome pathway analysis of differentially expressed genes in *ZNF451*-shRNA versus Ctrl-shRNA MRC5 cells. **C** Gene set enrichment analysis (GSEA) of pathways associated with fibroblast activation that were enriched in *ZNF451*-shRNA versus Ctrl-shRNA MRC5 cells. **D** Volcano plots show differential gene expression between Ctrl-shRNA and *ZNF451*-shRNA MRC5 cells. **E** RT‒PCR of *PDGFB* mRNA in Ctrl-shRNA and *ZNF451*-shRNA MRC5 cells. **F** RT‒PCR of *Pdgfb* mRNA in primary lung fibroblasts isolated from WT mice or *Znf451*-/- mice. **G** RT‒PCR of *Pdgfb* mRNA in lung tissues from WT mice or *Znf451*-/- mice. **H** GSEA showing that the PI3K-Akt signaling pathway is activated in *ZNF451*-shRNA vs. Ctrl-shRNA MRC-5 cells. **I** Correlation analysis between the mRNA expression of *Znf451* and *Pdgfb* in PF lungs (*n* = 9)

To explore the downstream signaling pathway regulated by ZNF451, we examined gene expression in Ctrl- versus *ZNF451*-silenced fibroblasts using RNA microarrays. A total of 771 differentially expressed genes (DEGs) were found in *ZNF451*-deficient cells, including 500 upregulated and 271 downregulated genes (Fig. 6A). Reactome pathway analysis showed that the differentially expressed genes were enriched in 79 specific pathways, among which several associated with activated fibroblasts were significantly enriched, including collagen formation, extracellular matrix organization, nonintegrin membrane-ECM interaction, collagen fibril assembly and other multimeric structures (Fig. 6B, C). Volcano plots exhibited the differentially expressed genes between Ctrl-shRNA and *ZNF451*-shRNA, among which *PDGFB*, a well-known profibrotic gene [22, 23], caught our attention (Fig. 6D). qPCR analysis showed that *ZNF451* knockdown efficiently increased *PDGFB* expression in MRC5 human fibroblasts (Fig. 6E). Similar results were observed in mouse primary lung fibroblasts (Fig. 6F). Additionally, higher *Pdgfb* expression was found in the lung tissues of BLM-exposed *Znf451*−/− mice than in those of BLM-exposed WT mice (Fig. 6G). PDGFB triggers the PI3K-Akt signaling pathway, which promotes the activation of fibroblasts into myofibroblasts [24, 25]. We reanalyzed the RNA sequencing data and found that ZNF451 deficiency activated the PI3K-Akt signaling pathway (Fig. 6H). In addition, a negative correlation of *Znf451* and *Pdgfb* expression was found in the lung tissues of PF mice (Fig. 6I). Collectively, these data suggest that ZNF451 deficiency might activate fibroblasts by increasing the expression of PDGFB and enhancing the PI3K-Akt signaling pathway.

**Fig. 7:**
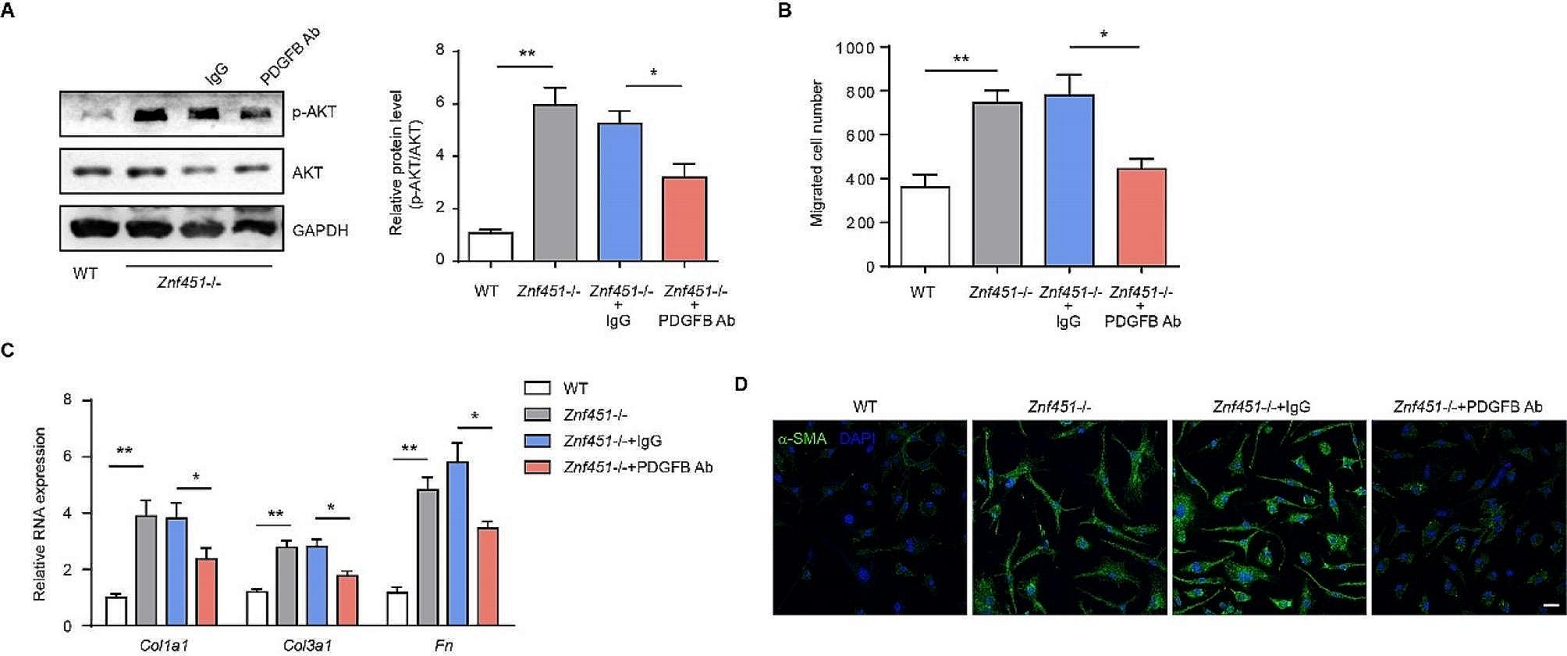
PDGFB-neutralizing antibody attenuates ZNF451 depletion-induced lung fibroblast activation. **A** Immunoblots of phosphorylated and total AKT in lung fibroblasts in response to PDGFB antibody stimuli. **B** Migration analysis of lung fibroblasts from BLM-challenged WT or *Znf451*-/- mice treated with or without PDGFB antibody. **C** RT‒PCR of mRNA expression of fibrosis genes in lung fibroblasts from BLM-challenged WT or *Znf451*-/- mice treated with or without PDGFB antibody. **D** Representative images of α-SMA immunostaining in lung fibroblasts from BLM-challenged WT or *Znf451*-/- mice treated with or without PDGFB antibody. Scale bars, 50 μm

We examined the potential effect of PDGFB-neutralizing antibody on ZNF451 deficiency-induced AKT signaling pathway and fibroblast activation. We therefore analyzed the phosphorylation of AKT by immunoblotting. We found that the enhanced AKT phosphorylation in *Znf451*-depleted fibroblasts was downregulated in response to PDGFB-neutralizing antibody (Fig. 7A). Additionally, blocking PDGFB with a PDGFB-neutralizing antibody almost completely abolished the effect of ZNF451 deletion on the enhanced migration capacity of fibroblasts (Fig. 7B). In addition, treatment with PDGFB-neutralizing antibody decreased the increased expression of these fibrosis-related genes in ZNF451-deleted fibroblasts (Fig. 7C). Immunofluorescence staining showed that the increased expression of a-SMA caused by ZNF451 depletion was reversed in lung fibroblasts treated with PDGFB-neutralizing antibody (Fig. 7D). Together, our results demonstrated that ZNF451 regulates fibroblast activation via PDGFB.

## Discussion

No effective therapies for PF exist due to the lack of effective therapeutic targets. In the present study, we provide several lines of evidence suggesting that the abnormal expression of ZNF451 plays a crucial role in PF. Lungs isolated from mice following BLM-induced PF were characterized by reduced ZNF451 expression. Importantly, a negative correlation was found between the expression of ZNF451 and fibrosis-related genes, such as COL1A1, COL3A1, and FN1. By using ZNF451-deficient mice, we found that ZNF451 depletion led to the exacerbation of PF in BLM-challenged mice. Consistently, overexpression of ZNF451 protected mice from BLM-induced pulmonary fibrosis. We further confirmed the dysregulation of ZNF451 in fibroblasts, which are the principal effector cells in PF. Our results support that strategies aimed at increasing ZNF451 expression in fibroblasts could be a viable therapy against pulmonary fibrosis in clinical settings.

A number of studies have reported that ZNF451 acts as a transcriptional cofactor to regulate target gene expression [18, 19]. For instance, ZNF451 positively regulates androgen signaling in prostate cancer cells by acting as a transcriptional coactivator for the androgen receptor [13]. Feng et al. reported that ZNF451 negatively regulates transforming growth factor β (TGF-β) signaling by acting as a transcriptional corepressor for Smad3/4 [18]. Furthermore, our recent study has shown that ZNF451 can act as a transcriptional coactivator for the snail family transcriptional repressor 2 (SLUG), thus enhancing SLUG-mediated CCL5 transcription [19]. Here, by using RNA sequencing, we found that ZNF451 knockdown in lung fibroblasts significantly increased the expression of PDGFB, a crucial profibrotic factor. Accordingly, functional analysis revealed enrichment of the PI3K-Akt signaling pathway, suggesting that the PDGFB/PI3K/Akt signaling pathway is involved in the ZNF451 knockdown-induced activation of fibroblasts into myofibroblasts. Indeed, treatment with PDGFB-neutralizing antibody ameliorated ZNF451 knockdown–induced fibroblast activation, confirming that ZNF451 regulates the activation of fibroblasts via PDGFB. Given that Feng et al. reported that ZNF451 acts as an inhibitor of the TGF-β/Smad signaling pathway [18], which also plays a crucial role in the activation of myofibroblasts and the progression of pulmonary fibrosis [26], whether ZNF451 regulates fibroblast activation through the TGF-β/Smad pathway needs to be clarified in future studies.

PDGF is widely implicated in the pathogenesis of PF [27, 28]. In human PF, the mRNA levels of the ligands PDGFA and PDGFB and the receptors PDGFRA and PDGFRB are upregulated in whole lung lysates [29]. Nintedanib, an FDA-approved drug for the treatment of PF, is a small molecule tyrosine kinase inhibitor (TKI) that binds to a family of growth factor receptors, including platelet-derived growth factor receptors (PDGFR), fibroblast growth factor receptors (FGFR), and vascular endothelial growth factor receptors (VEGFR) [30, 31]. Although nintedanib reduced lung function deterioration in patients with IPF in clinical trials, because tyrosine kinases are widely distributed in various cell types and participate in pleiotropic functions, patients receiving nintedanib treatment usually exhibit serious adverse events, including nausea, diarrhea, and liver dysfunction [32]. Further exploration of the therapeutic effects of specific targeting of PDGFB or its receptor PDGFRB in lung fibrosis will be of interest.

There are some limitations to our study. First, additional studies will be needed to investigate whether the dysregulation of ZNF451 expression also occurs in other cells, such as epithelial cells, endothelial cells, and macrophages, which also contribute to the pathogenesis of PF. Second, exploration of the reason why ZNF451 is downregulated in activated fibroblasts is warranted in future studies. Finally, ZNF451 may act as a transcriptional cofactor for PDGFB, and the inner mechanism underlying this process deserves to be further elucidated.

## Conclusions

In summary, we found that the reduced expression of ZNF451 in lung fibroblasts contributes to the progression of PF through upregulation of PDGFB expression, which drives fibroblast differentiation into myofibroblasts. Accordingly, genetically enhancing ZNF451 expression or neutralizing PDGFB with a PDGFB-neutralizing antibody may represent a novel therapeutic strategy for PF and other fibroproliferative lung diseases.

### Electronic supplementary material

Below is the link to the electronic supplementary material.

Supplementary Material 1

Supplementary Material 2

## Data Availability

Data will be made available on request.
