# Supplementary material for: Loss of ZNF451 mediates fibroblast activation and promotes lung fibrosis
Source: Respir Res. 2024 Apr 10;25:160. doi: 10.1186/s12931-024-02781-7 (PMC11008011; doi:10.1186/s12931-024-02781-7)
Supplement: Supplementary file 1 — Supplementary Material 1 [file 12931_2024_2781_MOESM1_ESM.docx]

**Table S1.** The primer sequences used for qRT-PCR.

| Gene |  | Primer Sequence (5’-3’) |
| --- | --- | --- |
| *ZNF451* | Forward | CTCTCTGTGCAAAGATGTTCCCT |
|  | Reverse | CAGCTACAGGTCCAGCATTTCG |
| *Znf451* | Forward | CAGCAAGACAGTGTGTGGACCA |
|  | Reverse | TGGCTTCTCAGACCTCCACATC |
| *PDGFB*  *Pdgfb*  *GAPDH*  *Col1a1*  *Col3a1*  *Fibronectin*  *Gapdh* | Forward  Reverse  Forward  Reverse  Forward  Reverse  Forward  Reverse  Forward  Reverse  Forward  Reverse  Forward  Reverse | GAGATGCTGAGTGACCACTCGA  GTCATGTTCAGGTCCAACTCGG  AATGCTGAGCGACCACTCCATC TCGGGTCATGTTCAAGTCCAGC  GTCTCCTCTGACTTCAACAGCG ACCACCCTGTTGCTGTAGCCAA  CCTCAGGGTATTGCTGGACAAC  CAGAAGGACCTTGTTTGCCAGG  GACCAAAAGGTGATGCTGGACAG CAAGACCTCGTGCTCCAGTTAG  CCCTATCTCTGATACCGTTGTCC  TGCCGCAACTACTGTGATTCGG  CCATCACTGCCACCCAGAAGACTG  ATGCCAGTGAGCTTCCCGTTCAG |
