# Supplementary figures and images for: Loss of ZNF451 mediates fibroblast activation and promotes lung fibrosis
Source: Respir Res. 2024 Apr 10;25:160. doi: 10.1186/s12931-024-02781-7 (PMC11008011; doi:10.1186/s12931-024-02781-7)

**Fig1.E**

ZNF451


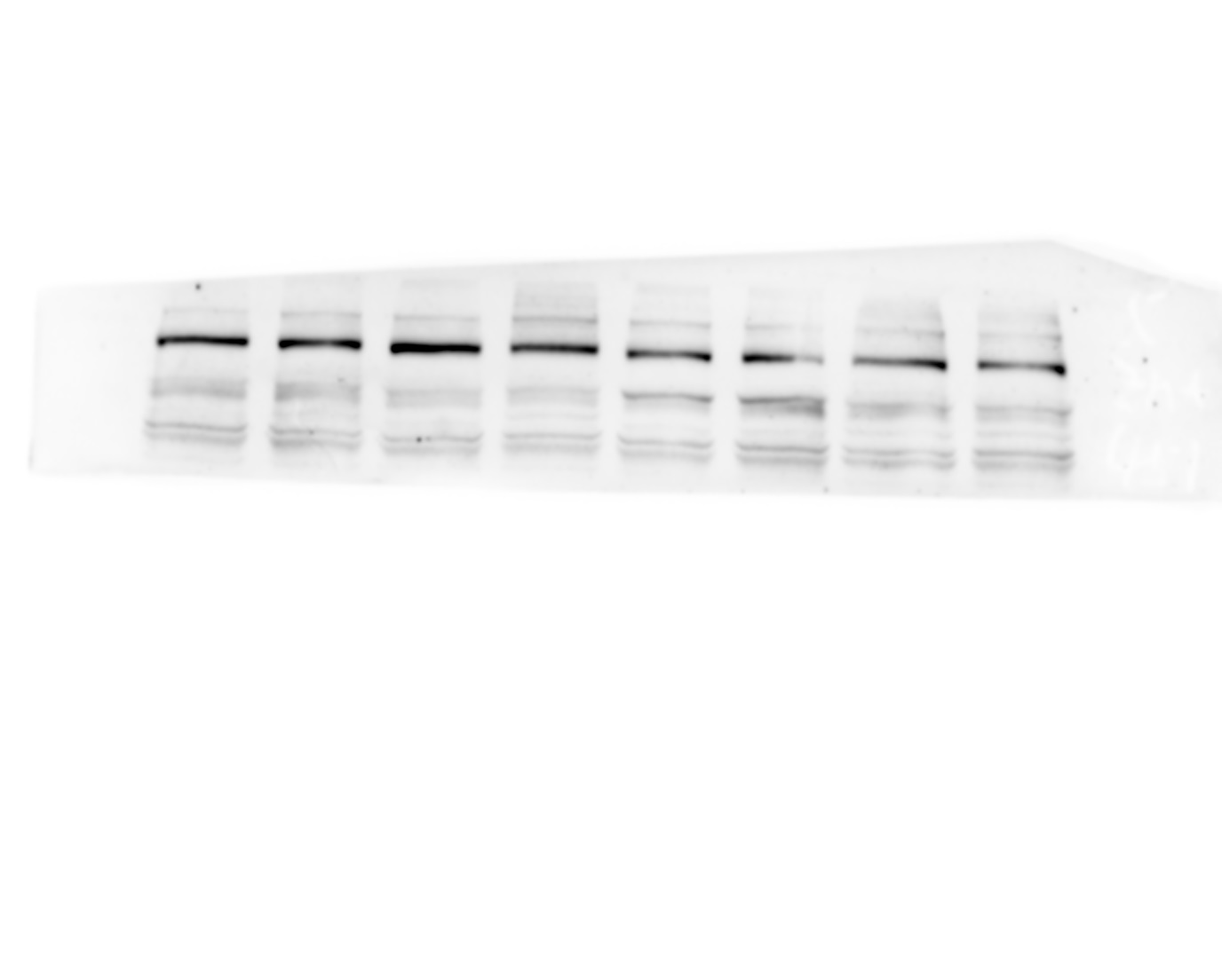


GAPDH


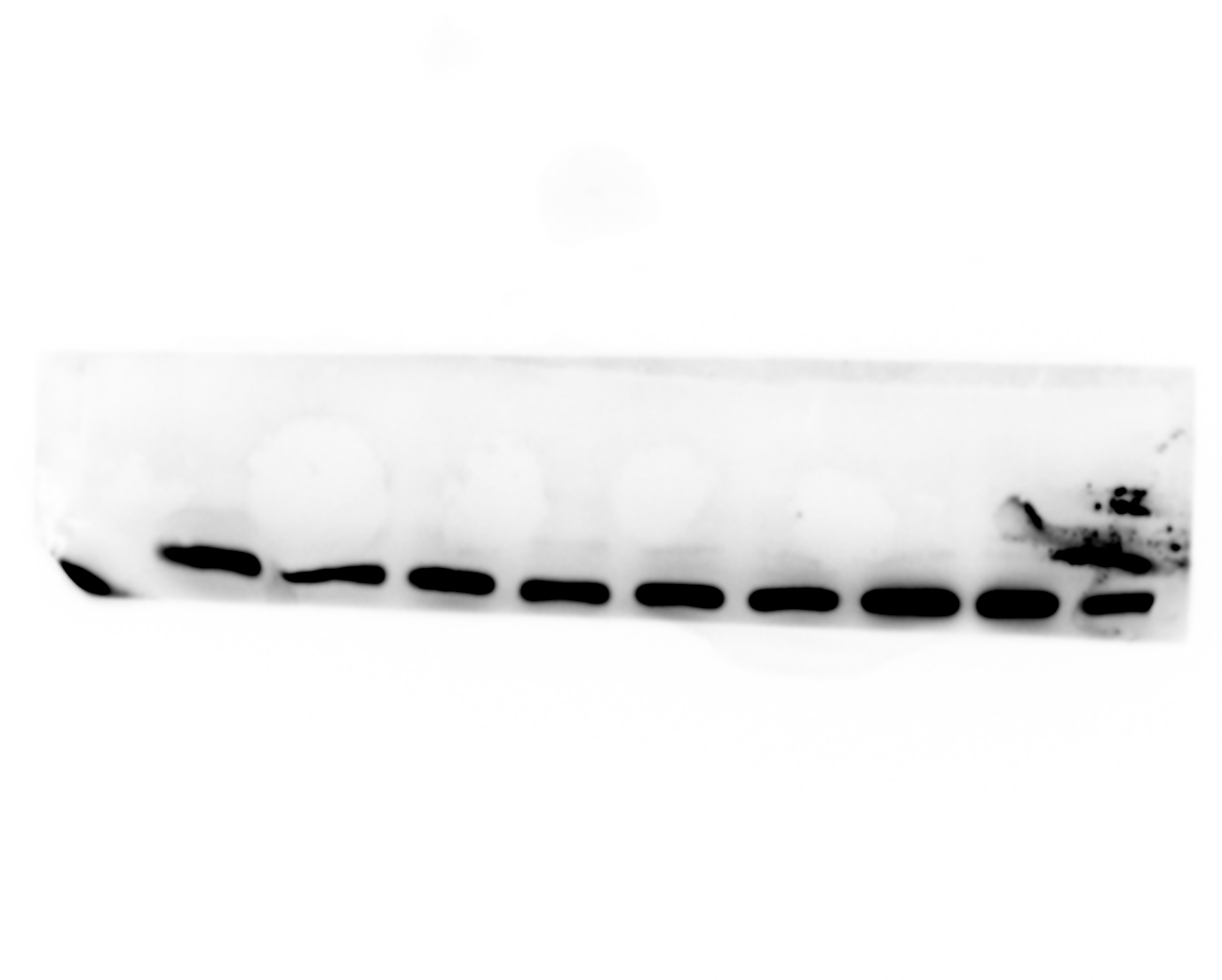


**Fig5.B**

a-SMA


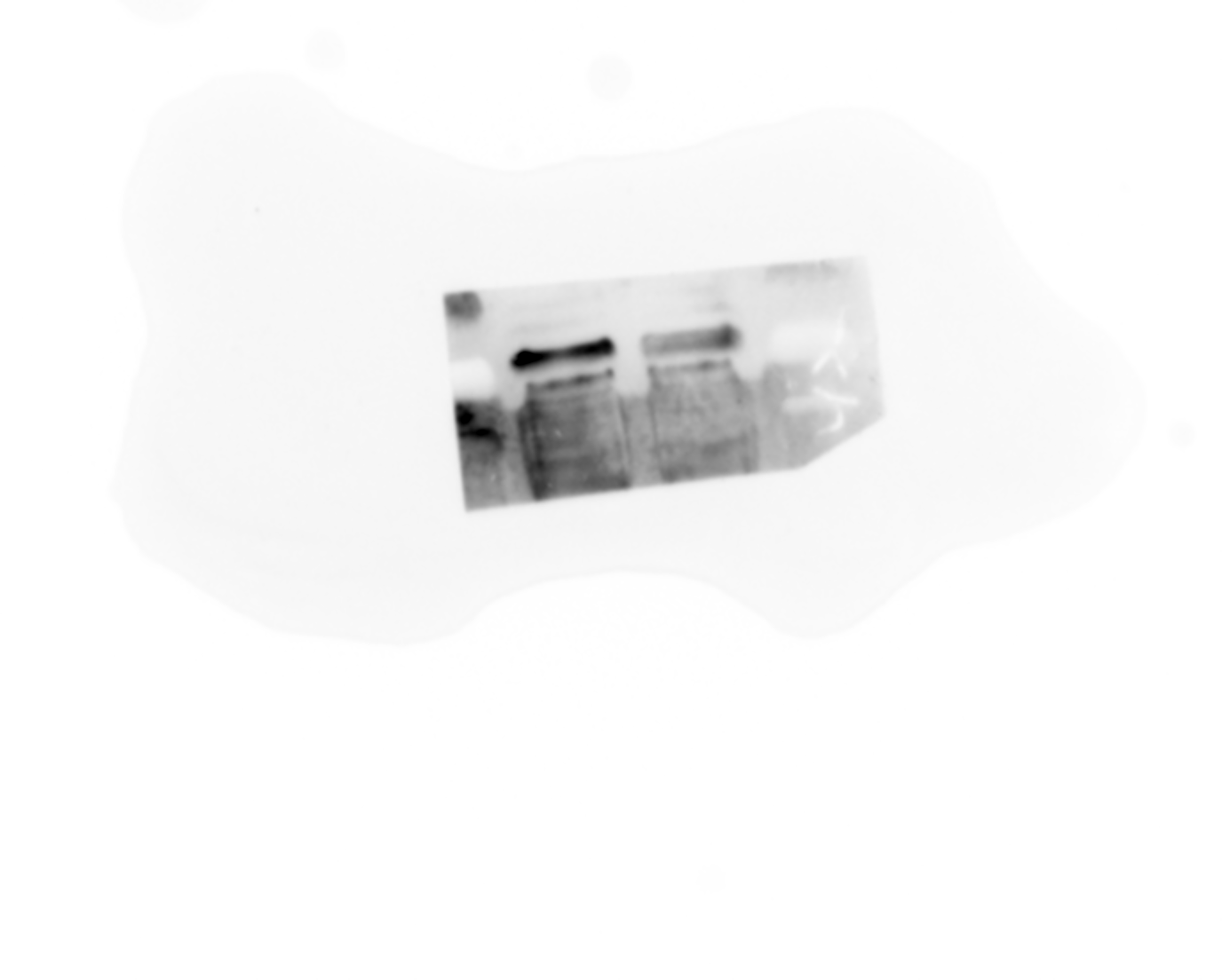


Col1


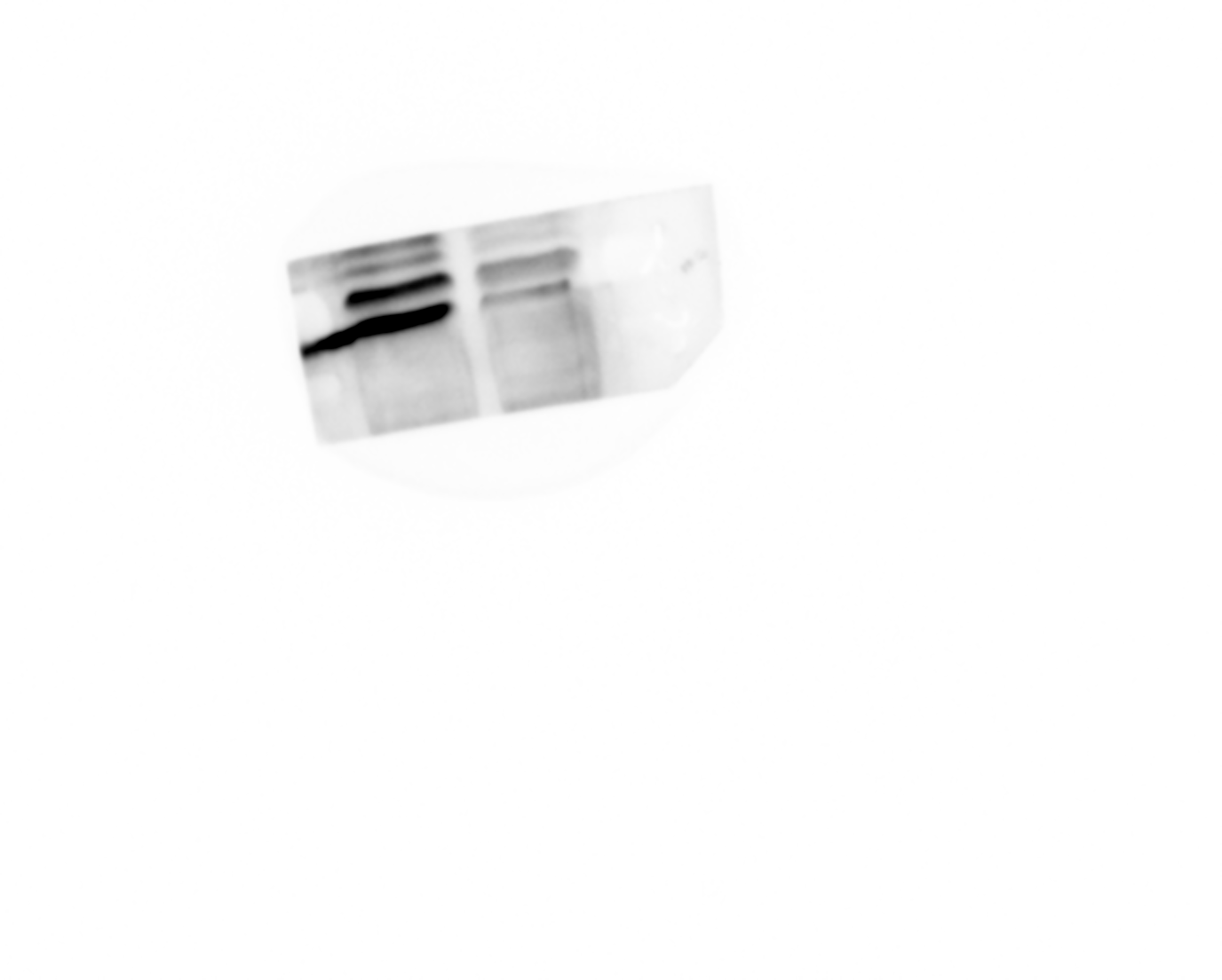


GAPDH


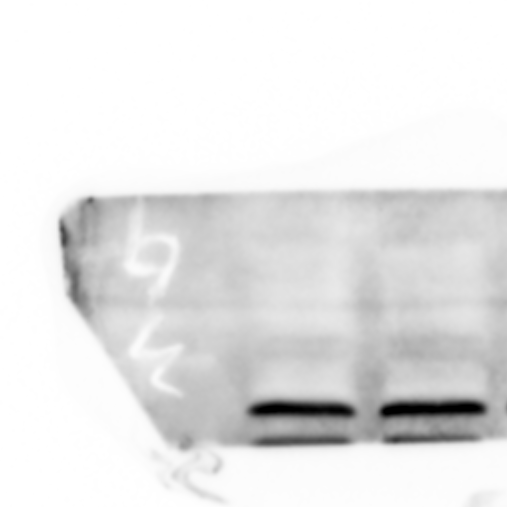


**Fig5.D**

a-SMA


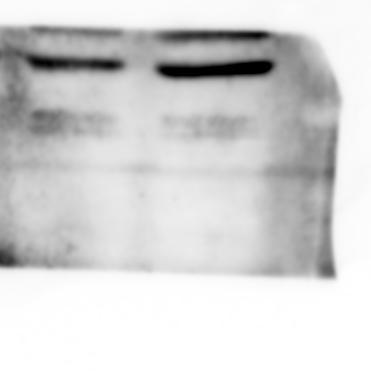


Col1


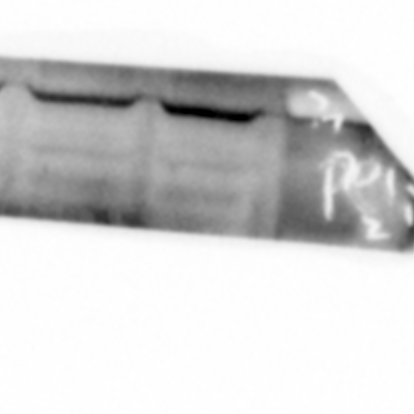


GAPDH


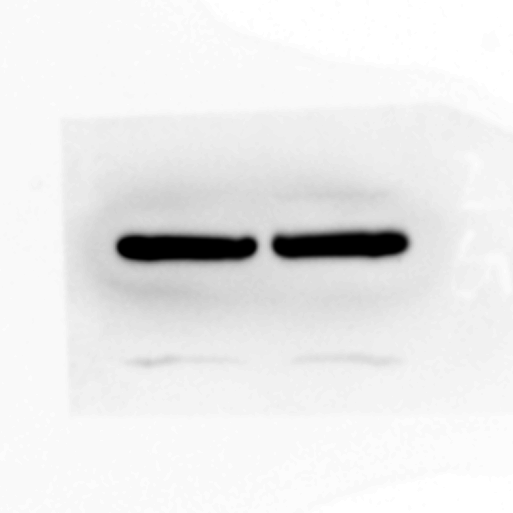

Supplement: Supplementary file 2 — Supplementary Material 2 [file 12931_2024_2781_MOESM2_ESM.doc]
